# Angiography and embolisation for solid abdominal organ injury in adults - a current perspective

**DOI:** 10.1186/1749-7922-5-18

**Published:** 2010-06-28

**Authors:** Adam Wallis, Michael D Kelly, Lyn Jones

**Affiliations:** 1 Department of Radiology, Frenchay Hospital, Frenchay Park Road, Bristol, BS16 1LE, UK; 2 Department of General Surgery, Frenchay Hospital, Frenchay Park Road, Bristol, BS16 1LE, UK; 3 Department of Radiology, Bristol Royal Infirmary, Upper Maudlin Street. Bristol, BS2 8HW, UK

## Abstract

Over the past twenty years there has been a shift towards non-operative management (NOM) for haemodynamically stable patients with abdominal trauma. Embolisation can achieve haemostasis and salvage organs without the morbidity of surgery, and the development and refinement of embolisation techniques has widened the indications for NOM in the management of solid organ injury. Advances in computed tomography (CT) technology allow faster scanning times with improved image quality. These improvements mean that whilst surgery is still usually recommended for patients with penetrating injuries, multiple bleeding sites or haemodynamic instability, the indications for NOM are expanding.

We present a current perspective on angiography and embolisation in adults with blunt and penetrating abdominal trauma with illustrative examples from our practice including technical advice.

## Introduction

Trauma is a leading cause of death and over 5 million people per year die from their injuries [1]. Patients often have abdominal injuries which require prompt assessment and triage. A recent study of over 1000 patients following abdominal trauma identified over 300 injuries on abdominal CT [2] and a study of 224 patients following abdominal trauma whom received CT regardless of haemodynamic stability identified 35 splenic injuries, 24 hepatic injuries and 13 renal injuries [3].

Emergency laparotomy is the standard treatment for patients with abdominal injury and haemodynamic instability. Over the past twenty years there has been a shift towards non-operative management (NOM) for haemodynamically stable patients without evidence of hollow viscus injury and, more recently for selected unstable patients [4]. The availability of rapid CT and the development and refinement of embolisation techniques has widened the indications for NOM in the management of trauma.

Optimal trauma management requires a multidisciplinary team, including surgeons and interventional radiologists, coupled with modern facilities and equipment. The emerging standard for trauma centres is the provision of multi-detector computed tomography (MDCT) within the emergency department [5] allowing rapid and complete CT diagnosis and improved clinical outcomes including reduction in ICU and hospital bed stays [6]. In addition there should be adequate provision of interventional radiology expertise - in practice this is not always the case.

Rapid assessment and treatment is vital in the management of patients with significant abdominal injury. Multiple bleeding sites or severe haemodynamic instability remain indications for surgery, and ATLS guidelines for the management of haemodynamically unstable patients advocate surgery without CT [7]. Patients who are stable or rapidly become stable with fluid resuscitation are suitable for CT, which will allow appropriate treatment decisions to be made. Traditionally a lot of time is spent on plain films but all of this information and more will be obtained by a CT. Embolisation aims to achieve haemostasis and salvage organs without the need for surgery, reducing the resuscitation period and transfusion requirements [8]. Super-selective embolisation is performed whenever possible.

This review gives a current perspective on the role of embolisation in adults with vascular complications arising from blunt and penetrating abdominal trauma, and includes illustrative examples from our practice and technical advice on 'how to do it'.

### Blunt and penetrating injuries to the abdomen

Protocols defining the role of transarterial embolisation in the management of the abdominal trauma victim vary among trauma centres, and many now advocate routine angiography [9]. There is substantial experience of embolisation in the management of blunt abdominal trauma, first described following hepatic injury in 1977 [10]. Splenic embolisation was initially described for haematological indications in the 1970s [11,12] and its use in the management of splenic injury was first reported in the early 1980s [13].

Angiography enables the identification and assessment of sites of haemorrhage. Angiographic embolisation of injured vessels has become a valuable adjunct in the management of trauma patients. It may provide life-saving haemostasis to areas that may be difficult to access surgically, prevent the need for re-operation in cases of rebleeding, or assist in the NOM of solid visceral injuries. Principles allowing the safe use of embolisation and NOM in blunt abdominal trauma include the absence of associated hollow visceral injuries and other injuries requiring operative intervention and lack of peritoneal signs on abdominal examination [14]. As experience increases, in the correct environment even haemodynamically unstable patients may be considered suitable for NOM [15].

The haemodynamic stability of the patient is key to management yet it is not easy to define. Shocked, unstable patients can be quickly identified and need rapid transfusion while urgent assessment and then treatment of the injury takes place. Stability implies repeated assessments over a period of time but it is usually abbreviated in patients with major abdominal trauma to initial response to fluid infusion. Haemodynamic stability may be defined as hemorrhagic shock not worse than Class 2, i.e. patients are normotensive, have elevated or normal pulse rate, respiratory rate <30/min, normal or decreased pulse pressure (arterial pulse amplitude), and have a rapid response to the initial fluid therapy of 2 L crystalloid [16]. The opinions of experienced clinicians should not be discounted in identifying quickly those patients which are most likely to deteriorate.

**Figure 1:**
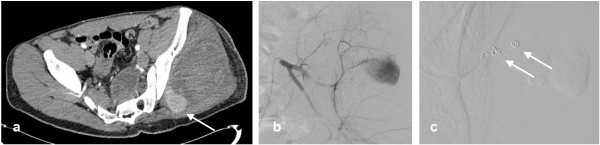
**a) Axial arterial phase contrast enhanced CT in a 23 year old man following a stab wound to the left buttock demonstrates haematoma within the gluteus muscles**. Contrast enhancement medially (arrow) represents active haemorrhage from the superior gluteal artery (Somatom sensation, 24 slice,Siemens, Erlangen, Germany). **b) **A Cobra catheter was negotiated into the posterior (somatic) left internal iliac artery from an ipsilateral approach. Active haemorrhage from a branch of the superior gluteal artery was demonstrated. **c) **A microcatheter system (Progreat) was negotiated into the bleeding vessel and 2 microcoils (Boston Scientific vortex fibred) were deployed (arrows). This completely abolished the bleeding with good perfusion of the buttock post procedure.

Experience with embolisation following penetrating truncal injuries is expanding. Velmahos demonstrated a success rate of 91% with embolisation used as a first line treatment, after operative failure to control bleeding or because of post-operative vascular complications [17]. The efficacy of embolisation at a number of sites within the abdomen was demonstrated, including the hepatic, internal iliac, renal, superior mesenteric and also gluteal vessels. Penetrating abdominal or pelvic trauma may also be associated with significant haemorrhage from non-visceral arteries as shown in figure 1.

The first large study of the use of embolisation in both blunt and penetrating abdominal trauma demonstrated a similar success rate of over 90% [18]. There was no difference between the success rates of embolisation for both. In over half the patients with penetrating trauma embolisation was used successfully after operative management failed to achieve haemostasis. The use of angiographic embolisation as a first-line treatment modality or as an adjunct to difficult surgery is supported by other studies [19].

### Interventional radiology techniques

In the context of expanding the role of NOM of abdominal trauma interventional radiology is used to control haemorrhage, either acutely or to prevent re-bleeding from pseudo aneurysms or in a post surgical patient.

The use of modern low osmolar contrast media (LOCM) for MDCT or angiography carries a small risk; mortality of 1 in 170,000 and severe or life threatening reactions of 1 in 40,000. In addition, if a patient has existing acute renal failure or severe chronic renal insufficiency, there is a risk of contrast induced nephropathy (CIN) of 5 to 50%. CIN is usually transitory and its significance is uncertain [20]. In the context of life threatening haemorrhage and in comparison to surgical morbidity for these patients, the risk of CIN would appear to be acceptable.

Occlusion balloons placed selectively and temporarily within internal iliac arteries, main visceral vessels or even within the aorta can be useful temporising measures. If there has been direct arterial trauma then assuming suitable anatomy stent graft or covered stent placement can provide a means to control the haemorrhage whilst preserving end organ blood supply. However, for solid organ haemorrhage embolisation is the most frequently used interventional technique.

Many different types of embolic materials are available (Table 1). Microcoils, delivered through coaxial microcatheters are the agents of choice if it is safe to effect permanent occlusion of a vessel and it has been possible to superselectively get close to the point of haemorrhage. In the renal circulation the vessels are end arteries and so it is usually sufficient to block the branch feeding the bleeding site. In the liver a rich collateral circulation means that this approach may not be ideal and embolising the vessels on both sides of the bleeding, so called 'closing the front and back door!' might be better. This can sometimes be achieved by passing beyond the bleeding point with the microcatheter and deploying a coil, then withdrawing proximal to the haemorrhage and deploying a second coil.

**Table 1:** Embolic materials

TEMPORARY	PERMANENT
GELFOAM SLURRY	COILS OR MICROCOILS (OFTEN FIBRED TO SPEED THE THROMBOTIC EFFECT)

AUTOLOGOUS CLOT	PARTICLES

	OCCLUSION DEVICES

	GLUE

	ONYX

If it proves impossible to obtain a superselective position close to the bleeding site then the choice is between proximal vessel embolisation with an occlusion device or larger coil to decrease haemostatic pressure at the bleeding site (good for splenic bleeding but prevents a second embolisation attempt if bleeding recurs) or the use of particles or gel foam to pass into the distal circulation, blocking smaller vessels. Use of particles runs a higher risk of ischaemic damage than superselective coil embolisation and therefore a temporary agent is often preferable. If using particles then larger sizes (500 μm diameter) are preferred as this leaves the capillary bed the potential to revascularise later from collaterals.

**Figure 2:**
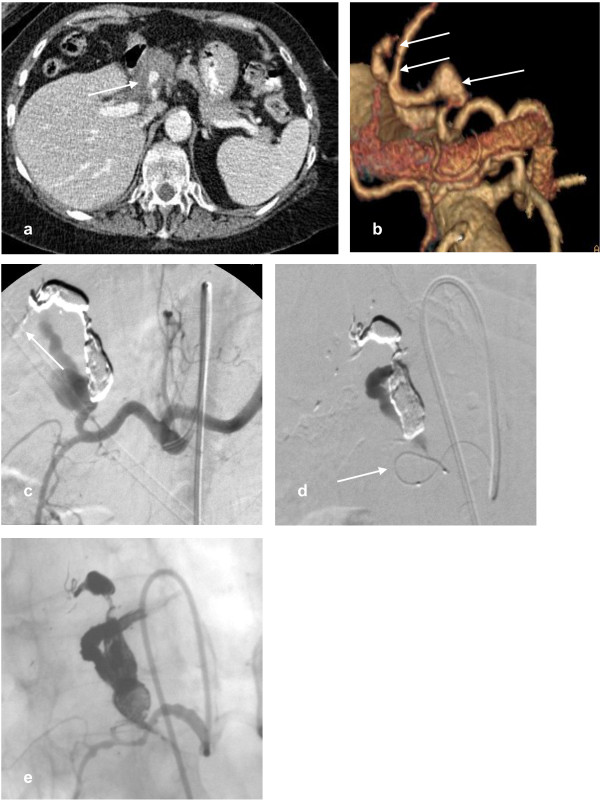
**a) A patient with vasculitic hepatic artery aneurysms presented following minor trauma**. Axial contrast enhanced CT demonstrates haematoma around a pseudoaneurysm (arrow) indicating that this is the likely cause of recent haemodynamic instability. **b) **3D volume rendered reconstruction demonstrates 3 aneurysms arising from a branch of the left hepatic artery (arrows). The right hepatic artery arose from the SMA. **c) **Selective arteriogram of the coeliac axis with standard catheter after 2 aneurysms had been embolised with onyx (ev3, Irvine, CA, USA). The cast of the onyx is demonstrated, and some distal embolisation (arrow) of onyx. **d) **A microcatheter is demonstrated within the final bleeding aneurysm (arrow). **e) **A selective angiogram demonstrates onyx filling all aneurysms and maintained patency of the gastroduodenal artery.

Onyx (ev3, Irvine, California, USA) is a polymer dissolved in dimethyl sulphoxide (DMS0) which is delivered as a liquid but becomes solid when in contact with blood. It takes time to prepare and deliver and is therefore less useful in the acute situation, but in the context of prevention of delayed haemorrhage it can be extremely useful as it can be deployed from a microcatheter proximal to a target. From the point of injection it will follow even tiny vessels distally to fill a pseudo aneurysm and continue on beyond, shutting both front and back doors without necessitating manipulation through the lesion with a microcatheter and wire. Figure 2 demonstrates embolisation of multiple hepatic artery aneurysms with onyx.

In practice, coils, microcoils and gelfoam slurry are the most common agents employed but availability of the full range of techniques is necessary in the delivery of an interventional trauma service.

## Splenic injuries

The spleen is the most commonly injured organ in severe abdominal trauma [21,22] particularly following blunt trauma [23]. To preserve immunological and haematological function and reduce the risk of post-splenectomy sepsis all attempts should be made to preserve the spleen. Following the acceptance of NOM in paediatric surgical practice the indications for NOM in adults have increased over the past 2 decades in an attempt to avoid the morbidity of surgery.

Several historic predictors of failure of conservative management, including complex splenic injuries [24], older age [25], pre-existing splenic pathology [26] or blood transfusion requirement are no longer universally accepted as reasons to avoid NOM of splenic trauma.

NOM has become the standard of care for haemodynamically stable patients, with failure rates of observational treatment reported as low as 5% [27]. Techniques include radiological intervention and careful monitoring.

i) CT imaging and classification of injury

CT is the imaging modality of choice in the evaluation of splenic injuries. With continued technical advances of CT scanners the CT can no longer be perceived as the 'doughnut of death' engendered by slower 1^st ^and 2^nd ^generation scanners. MDCT scanners have rapid diagnostic capability with increased spatial and temporal resolution [28] and should be considered a crucial step in the diagnostic pathway for stable patients.

CT has an accuracy of up to 98% in diagnosing acute splenic injuries [29]. CT grading correlates strongly with the actual injury seen at operation [30]. A recent study correlating MDCT with splenic arteriography noted an overall accuracy at detecting vascular injury of 83% [31]. Importantly, not all vascular injuries were detected prospectively on MDCT imaging and so angiography may still be necessary in high-grade injuries.

The American Association for the Surgery of Trauma organ injury scale (OIS) for the spleen, based on surgical appearance is widely referred to in the literature and clinical practice (Table 2).

**Table 2:** Spleen organ injury scale. [75]

I	Haematoma Laceration	Subcapsular, <10% surface area Capsular tear, <1 cm parenchymal depth
II	Haematoma Laceration	Subcapsular, 10% to 50% surface area; intraparenchymal, <5 cm in diameter Capsular tear, 1 cm to 3 cm parenchymal depth that does not involve a trabecular vessel

III	Haematoma Laceration	Subcapsular, >50% surface area or expanding; ruptured subcapsular or parenchymal haematoma; intraparenchymal, haematoma >5 cm or expanding >3 cm parenchymal depth or involving trabecular vessels

IV	Laceration	Laceration involving segmental or hilar vessels producing major devascularisation (>25% of spleen)

V	Haematoma Laceration	Completely shattered spleen Hilar vessel injury devascularises spleen

The accuracy of CT diagnosis depends on technique, and problems can arise with misdiagnosis and misgrading. Some patients with apparently low grade injury will still fail NOM, and CT is a morphological snapshot at a certain point in time and not an accurate predictor of subsequent haemorrhage [21]. Hence methods of grading the injury cannot be accurately used to distinguish patients at risk of delayed complications [32] and the use of splenic injury grade as the sole criterion for determining management strategy remains controversial [31].

CT grading systems incorporating MDCT findings of vascular lesions and active bleeding when assigning grade of injury have been suggested [33,34] and may be better than the AAST system for predicting which patients need angiography or intervention after blunt splenic trauma [35]. To date these are not in widespread use.

Indicators of the need for intervention in the form of transarterial embolisation or surgery include active contrast extravasation from the splenic parenchyma and vascular injuries such as pseudoaneurysm or arteriovenous fistula. At CT, these are demonstrated as an intraparenchymal contrast blush - a focal hyperdense collection of contrast. The presence of haemoperitoneum can also suggest vascular injury [31]. If the patient is hypotensive, parenchymal enhancement is often delayed and heterogenous and so appropriate CT technique with plain, arterial and delayed (2-3 minutes) phases of examination is necessary to achieve optimum sensitivity.

ii) Conservative management

The majority of blunt splenic injuries can be managed safely with observation, even in centres with a low incidence of trauma [36]. Embolisation is required in only 7% of patients [37] and conservative treatment of low grade injuries is successful in over 90% of patients [26,38].

Patients with a high grade injury are at greatest risk of failure of observational management (up to 70%) [25,26,30,38] and are at greatest risk of delayed operative intervention [14]. The need for transfusion of greater than 1 unit of blood is another independent risk factor for failure of observation [27,30] and haemodynamic instability will also determine further treatment as is discussed later.

Vascular injury (haemorrhage, haematoma, pseudoaneurysm or arteriovenous fistula) at CT is also associated with failure of observational treatment [26,32,39]. A contrast blush at CT scanning is associated with failure of observational treatment in up to 80% [32,39].

iii) The role of embolisation

Surgery is necessary if there is parenchymal destruction and injury to hilar vessels [40] an injury involving multiple vessels, associated hollow viscus injury or other injuries requiring operative intervention.

There are no set criteria to select patients for angiography and embolisation. If there is active bleeding (contrast blush) or non-bleeding vascular injury such as pseudoaneurysm, high grade injury or haemoperitoneum on CT, angiography is indicated [29,41,42]. Patients undergoing standard NOM in one study had volumes of haemoperitoneum approximating to blood in the perisplenic and/or perihepatic region and/or Morrison's pouch, whereas those undergoing angiography and embolisation had larger volumes with blood tracking down one or both paracolic gutters and in some patients into the pelvis [41]. Arterial extravasation detected by MDCT is present in between 13% and 17.7% of patients [21,22]. Extravasation has a high sensitivity in predicting the need for angiography and subsequent endovascular treatment or splenic surgery [21,29].

If angiography confirms active bleeding, embolisation should be performed. Dent et al expanded the role of embolisation to include significant haemoperitoneum, grade 4 or 5 splenic injury, decreasing haematocrit not explained by other injuries, and persistent tachycardia [37].

Whilst haemodynamic instability is difficult to define, it has historically been an indicator for surgical intervention [30]. This is now controversial with some studies demonstrating safe effective use of embolisation in unstable patients. In one study, patients with a systolic blood pressure of <90 mmHg and shock index (heart rate divided by systolic blood pressure) of >1.0, and a transient response to fluid resuscitation underwent angiography [15]. Whilst only 15 patients were included (mean systolic blood pressures of 84.2 mmHg), embolisation was successful in all, with no reported complications. Other studies demonstrate rapid normalisation of haemodynamic status as would be expected in haemodynamically unstable patients following embolisation [41]. Ultimately the decision will depend on local experience and service availability.

Many authors have used embolisation as an adjunct to NOM [42-44]. Success rates of NOM in high grade injuries of 95% have been documented with this strategy [45]. Splenic artery embolisation in selected patients without evidence of active bleeding is a safe and useful adjunct to NOM [37,41]. Some authors have expanded the indication for angiography to include some patients without contrast blush on CT. Gaarder et al., demonstrated increased success rates of NOM from 79% to 96% when mandatory angiography (and embolisation if indicated) was performed on all high grade injuries (with a high rate of failure of NOM and risk of delayed bleeding) regardless of the presence of contrast blush [46]. The splenic salvage rate increased with fewer complications of delayed bleeding compared to historical controls when mandatory angiography was not performed on all high grade injuries.

**Figure 3:**
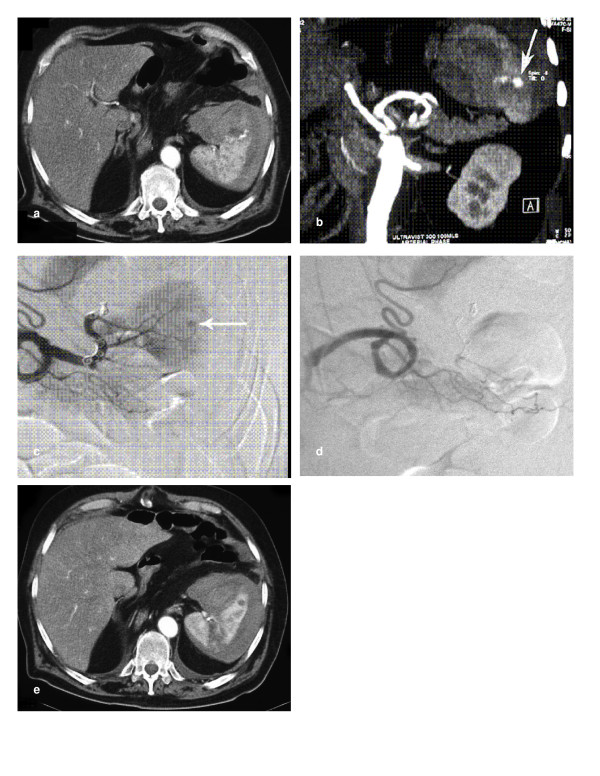
**a) Axial CT of a 73 year old man with iatrogenic splenic injury following chest drain insertion**. An active bleeding point in the spleen (arrow) with surrounding haematoma was demonstrated. **b) **Coronal CT reconstruction showing a tortuous splenic artery and bleeding point (arrow). These allowed optimal catheter choice for arteriography. **c) **A Tracker-18 microcatheter system with a Fasdasher 0.014 in wire (Boston Scientific, Maple Grove, MN, USA) were used to achieve access distally within the splenic circulation. After several unsuccessful attempts at superselective catheterisation of the branch supplying the bleeding point, 4 platinum Vortex-18 diamond-shaped coils (Boston Scientific) were deployed sequentially in the main splenic artery distal to the dorsal pancreatic branch. 2 initial coils migrated past the required branch and there is ongoing bleeding from the spleen (arrow). **d) **The next 2 coils achieved occlusion of the main splenic artery with preservation of branches to the dorsal pancreas and upper pole of the spleen. **e) **Axial CT at 1 week showed a small splenic infarct where the initial coils had migrated distally. Arterial supply to the spleen was preserved with some flow through the main splenic artery coils.

Superselective embolisation of the bleeding segmental artery using microcatheter techniques when possible may ensure a greater likelihood of the immune function of the spleen remaining uncompromised [47] though may be associated with increased complication rates [48]. Benefits of preserving splenic function must be balanced against the risk of delayed haemorrhage even in patients with low grade injuries [29,32]. CT reconstructions as shown in figure 3 can help to guide catheter selection by providing a 'roadmap' of the splenic artery [49].

iv) Complications of embolisation

Recent studies report failure rates for embolisation as low as 2.7% to 4% [41,46] after proximal embolisation for high grade lesions, active contrast extravasation or haemoperitoneum. However, proximal rather than selective embolisation may result in fewer complications [48] and other studies have recorded a higher overall complication rate for embolisation of around 27% [50,51]. Patient selection is therefore considered crucial and the authors highlight the necessity for a low threshold for further intervention if there are signs of continued bleeding post-embolisation.

A retrospective study comparing embolisation to operation demonstrated a significantly lower number of complications in the embolisation group (13%) than the operative group (29%) [27]. The complications attributed to embolisation are generally minor and need to be viewed in the context of having avoided an operation with its attendant morbidity.

Minor complications can be expected in up to half if fever is included [45] and fever and reactive pleural effusion can be considered as a form of mild post-embolisation syndrome. Infarcts may occur in up to 20% of patients (more so with distal embolisation) but usually resolve without clinical sequelae [52]. Recurrent haemorrhage can occur in up to 11% and abscess in 4%. Coil migrations and splenic artery dissections are potential but rarely encountered complications [41].

## Hepatic injuries

The liver is frequently injured following abdominal trauma and is often associated with splenic injury [53]. Most liver injuries heal spontaneously and conservative management is safe for haemodynamically stable patients with hepatic injury regardless of severity [51].

i) CT imaging and classification of injury

CT can accurately determine the location and extent of hepatic injury and demonstrate intra- or extra-hepatic haemorrhage. It is an important factor in allowing safe NOM of hepatic injuries [54]. Patterns of injuries include capsular tear, parenchymal laceration or fracture, subcapsular and intraparenchymal haematoma and partial devascularisation due to parenchymal injury. The American Association for the Surgery of Trauma organ injury scale for the liver is shown in Table 3 though again this may underestimate injury severity and includes some criteria that cannot be assessed by CT.

**Table 3:** Liver organ injury scale. [75]

I	Haematoma Laceration	Subcapsular, <10% surface area Capsular tear, <1 cm parenchymal depth
II	Haematoma Laceration	Subcapsular, 10% to 50% surface area; intraparenchymal, <10 cm in diameter Capsular tear, 1 cm to 3 cm parenchymal depth, <10 cm in length

III	Haematoma Laceration	Subcapsular, >50% surface area of ruptured subcapsular or parenchymal haematoma; intraparenchymal, haematoma >10 cm or expanding >3 cm parenchymal depth

IV	Laceration	Parenchymal disruption involving 25% to 75% hepatic lobe or 1 to 3 Couinaud's segments

V	Laceration Vascular	Parenchymal disruption involving >75% of hepatic lobe or >3 Couinaud's segments within a single lobe Juxtahepatic venous injuries, ie retrohepatic vena cava/central major hepatic veins

VI	Vascular	Hepatic avulsion

**Figure 4:**
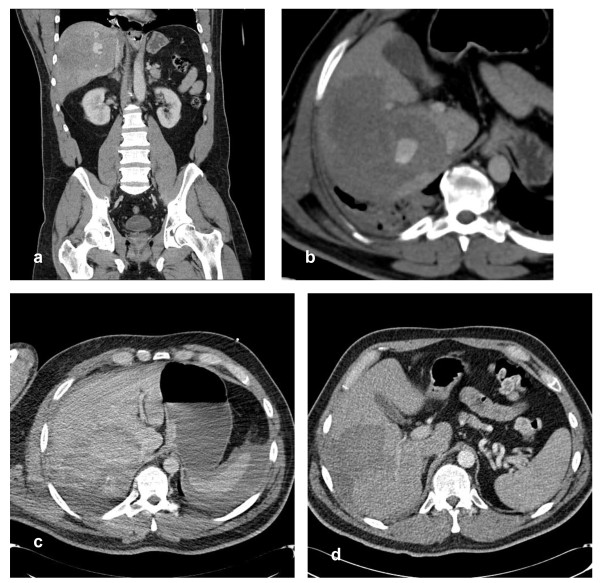
**a) Coronal contrast enhanced arterial phase CT reconstruction showing contrast blush in a contained right lobe haematoma due to blunt inury**. **b) **Axial CT demonstrates the blush. **c) **Scan at 18 hours showing no blush but capsular rupture with intraperitoneal blood. **d) **Follow up CT at 9 weeks showing resolving right lobe haematoma.

High quality CT is critical to the management of the patient with a major liver injury because of the dual vascular inflow. A contrast blush could represent portal venous rather than arterial bleeding on a non-arterial phase scan. The absence of contrast blush and hepatic vein involvement is considered the most reliable CT evidence to exclude active bleeding. An arterial contrast blush from a major blunt liver injury is shown in figure 4. The liver capsule was intact and angiography with a view to selective embolisation was not performed because of a decision by the oncall surgeon. CT scan 18 hours later showed no active bleeding; however there was free intraperitoneal blood consistent with capsular rupture which may have been avoided by embolisation.

ii) Conservative management

Multiple studies have demonstrated effective conservative management of blunt and penetrating liver injuries [41,24,55,56]. Whilst grade of injury initially was believed to be predictive of the need for operative intervention, even high grade injuries (IV-V) have been successfully managed without surgery and vascular injuries can be managed with radiological intervention.

Additional treatment due to complications may be required in between 13.5% [53] and 24% [57] of patients. Bile leak is frequently encountered and a large proportion (up to 25%) of patients require percutaneous interventional techniques to drain bile collections some of which go on to form a biliary fistula which may require endoscopic stenting [58]. Other complications observed during conservative treatment of blunt hepatic injuries include biloma formation, arteriovenous fistula or pseudoaneurysm formation and abscess formation [59]. Nonoperative interventional procedures can be used to treat complications that arise during the course of conservative treatment of liver injury in up to 85% [57].

Haemodynamically stable patients without CT evidence of extravasation can be managed conservatively, even in the presence of extensive parenchymal injury [59]. Figure 2 demonstrates the embolisation of multiple hepatic artery aneurysms using onyx.

Intrahepatic vascular lesions may accompany high grade injury, and extension of injury into the main trunk of one or more hepatic veins is an indicator that conservative management will fail. NOM is also more likely to fail in patients requiring more blood transfusions and with higher injury severity scores [56].

iii) The role of embolisation

Active extravasation is encountered less than splenic injury (in only 9.1% of patients [22] but still correlates with need for active management with 81% of these patients requiring surgery or embolisation [21]. Embolisation offers an effective way for early control of bleeding in the presence of a contrast blush, and should be used as a valuable adjunct to NOM [18,19]. Velmahos et al. reserved angiography for urgent haemostasis after damage control operations or for signs of active extravasation on the CT scan. This increased success rates to 85% with a liver-specific success rate of 100% [56]. Other studies have demonstrated similar or better success rates with embolisation [60,61].

Haemodynamic instability was regarded until recently as one of the best predictors of the need for operative management [51]. As with splenic injuries there is increasing experience with embolisation in these high risk patients. A multidisciplinary approach with a role for embolisation even in haemodynamically unstable patients achieved a success rate of 93% in one recent study [62]. 3 patients required over 2 L/h of fluid resuscitation and underwent early angiography and selective embolisation with good results. 8 patients with high grade injury and a mean transfusion requirement of 5.6 units (range 2-11) also had a good result. Perihepatic packing at laparotomy was used to stabilise 4 separate patients prior to successful embolisation. Ultimately the use of embolisation in haemodynamically unstable patients depends on the clinical scenario and local experience.

**Figure 5:**
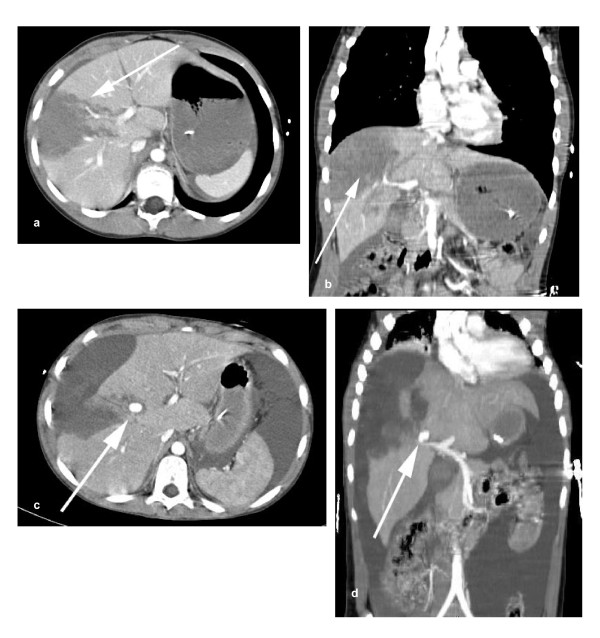
**a) Axial contrast enhanced CT of a teenager who sustained a handlebar injury to the abdomen**. Large laceration/haematoma (arrow) and no active extravasation. **b) **Coronal reconstruction demonstrates free fluid around the right lobe of the liver (arrow) and the extent of the laceration. He was managed conservatively initially but deteriorated several days later. **c) **An emergency CT showed a contrast blush (arrow). **d) **Maximimum intensity projections demonstrated that the most likely cause was the right anterior portal vein (arrow). At operation (not by our team) biliary peritonitis was found but there was no active bleeding and subsequent hepatic angiography was negative.

In contrast to splenic injuries, delayed bleeding from the liver in blunt trauma is reported to be rare [63]. However it is the most common vascular complication of NOM of liver injuries, occurring in up to 3% of patients [55]. A change in the haemodynamic status of any patient having NOM of an abdominal injury mandates urgent CT scan. Figure 5 shows a grade III liver laceration that was initially treated conservatively but the patient required delayed operative management due to clinical deterioration. Complications such as false aneurysm or a posttraumatic arterio-portal fistula are more likely following penetrating injury and are amenable to embolisation [64].

Angiographic related complications are infrequent and as low as 0% [62] though other studies have shown that up to 14% of patients may require re-embolisation due to continued bleeding [56]. Reported complications include; bile collections, hepatic abscess, gallbladder infarction and subcapsular haematoma. Some of these are not a direct result of embolisation but of NOM and the trauma itself [62].

Follow-up CT is warranted for monitoring of NOM of all major hepatic injuries in order to enable early detection of complications such as A-V fistula.

## Renal injuries

Renal injuries may occur after stab and gunshot wounds but are more common after blunt abdominal trauma or iatrogenic following percutaneous renal procedures. Renal trauma comprises up to 24% of injuries resulting from blunt abdominal trauma, third only to splenic and hepatic injuries [65]. Most (over 80%) can be considered minor and heal [66]. Renovascular injuries occur in only 2.2% of all patients with blunt abdominal traumatic injuries [66].

The range of CT appearances includes contusions (seen as ill-defined perfusion defects), superficial lacerations, segmental renal ischaemic infarcts (seen as segmental perfusion defects) and subcapsular or perirenal haematoma. Evaluation of renal injuries requires standard parenchymal phase imaging and delayed nephrogenic phase imaging giving information on the collecting system [40]. This will help differentiate contrast extravasation from the renal pelvis (posttraumatic urinoma) from active haemorrhage from the renal parenchyma.

The American Association for the Surgery of Trauma organ injury scale for the kidney is shown in Table 4. There is a significant association between renal injury severity as assessed by this classification and the potential for developing permanent parenchymal scarring on follow up CT scans [67].

**Table 4:** Kidney organ injury scale. [75]

I	Contusion Haematoma	Microscopic or gross haematuria, urologic studies normal Subcapsular, nonexpanding without parenchymal laceration
II	Haematoma Laceration	Nonexpanding perirenal haematoma confined to renal retroperitoneum <1 cm parenchymal depth of renal cortex without urinary extravasation

III	Laceration	>1 cm parenchymal depth of renal cortex without collecting system rupture or urinary extravasation

IV	Laceration Vascular	Parenchymal laceration extending through renal cortex, medulla and collecting system Main renal artery or vein injury with contained haemorrhage

V	Laceration Vascular	Completely shattered kidney Avulsion of renal hilum that devascularises kidney

Conservative management is the usual approach for renal injuries in the absence of haemodynamic instability. Most will heal spontaneously and tamponade by the retroperitoneal fascia limits renal bleeding. Avulsion of the renal pelvis and injury of the vascular pedicle are accepted indications for surgery [68]. Trauma-induced pseudoaneurysm, massive haemorrhage or continuous haematuria also suggest the need for more aggressive therapy [69].

**Figure 6:**
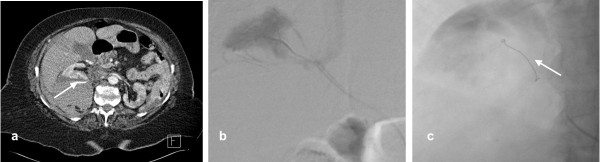
**a) A 76 year old lady on warfarin presented with abdominal and back pain following a fall**. Contrast enhanced axial CT demonstrates retroperitoneal haematoma associated with a ruptured right kidney and evidence of active contrast extravasaion (arrow). **b) **Selective catheterisation of the right kidney showed a bleeding focus in the upper pole. **c) **The branch to the upper pole was selectively catheterised and embolised using a single platinum coil (arrow). Post procedure renal arteriogram demonstrated cessation of haemorrhage.

Studies have described the utilisation of renal arterial embolisation in renal trauma [69]. Figure 6 illustrates the use of embolisation to treat active renal extravasation. Arterial lacerations and ruptures, arteriocalyceal fistulae, pseudoaneurysms and arteriovenous fistulae are the most common renal vascular injuries [70]. The latter two usually occur secondary to penetrating trauma. Delayed bleeding after surgery or trauma is not uncommon and significant bleeding is associated with angiographically identifiable lesions in the majority of cases [71].

In haemodynamically stable patients with vascular injury the treatment of choice is percutaneous selective embolisation which is directed to the site of injury by a previously performed CT examination [40]. Sofocleus et al., performed selective or superselective embolisation in patients following blunt or penetrating abdominal trauma with immediate technical success in 91%. The indications were CT examination consistent with arterial injury, intraoperative findings such as haematoma with possibility for angiographically assisted renal salvage, and signs/symptoms suggestive of vascular renal injury [70].

Renal pedicle vascular injuries are rare and occur in 1 to 4% of renal injuries. They are usually managed surgically though patients with traumatic renal artery dissection may be treated with endovascular stent placement, made possible with early CT diagnosis [72]. Patients with high grade injuries not involving the vascular pedicle but with CT findings consistent with active haemorrhage have been successfully managed with embolisation [69]. A recent 10- year review of the use of intervention in renal vascular injury demonstrated a success rate of over 94% in patients undergoing angiography and embolisation as primary management (34.4% of patients) [73]. A further 23% of patients were managed conservatively and all those that required primary laparotomy did so for life-threatening haemorrhage or associated injuries.

Technical failures requiring repeat angiography and embolisation can occur in up to 9.5%, and renal abscess in up to 5% [70]. Other rare but potential complications of renal embolisation include contrast nephropathy, renal infarction and haemorrhagic shock induced acute renal injury. With selective embolisation, the extent of a renal infarct can be significantly reduced resulting in excellent preservation of functioning renal tissue [70]. The choice of treatment depends on the condition of the patient and their injury, and the availability of interventional services. Superselective embolisation of renal artery branches is also the treatment of choice following iatrogenic trauma to the kidney [74].

## Conclusion

There is a paucity of good quality evidence for use of MDCT and/or embolization in trauma patients who are not completely stable consequently there is currently wide variation in practice with regard to the inclusion of angiography within treatment algorithms, both within the UK and worldwide [4]. There is a need for greater access to MDCT and interventional radiology facilities including sufficient numbers of appropriately trained interventional radiologists and support staff to provide 24 hour cover at trauma centres. Once the infrastructure is in place prospective multicentre trials can be designed to determine optimum future treatment algorithms. Until then practice depends upon local facilities and availability and experience of surgeons and radiologists.

NOM is now the treatment of choice for abdominal trauma with solid organ injury. Significant hollow organ or pancreatic injury is generally an indication for surgical management. Embolisation has an accepted role as an adjunct to NOM of abdominal trauma in haemodynamically stable patients with a contrast blush seen on arterial phase CT. It also has a role in the treatment of bleeding complications following operative intervention. Its application has been limited until now by patchy provision of emergency interventional radiology and the perception of the CT scanner as the 'doughnut of death'. The emerging standard for centres involved in the management of trauma is the provision of state of the art MDCT within the emergency department and 24 hour availability of interventional radiology. This will allow rapid diagnosis by CT and treatment by interventional radiology of patients traditionally treated by emergency laparotomy because of haemodynamic instability. The challenge for emergency physicians, surgeons and radiologists is to put this system in place for the safe non-operative management of tomorrow's abdominal trauma patients.

## Competing interests

The authors declare that they have no competing interests.

## Authors' contributions

LJ and MK conceived the review. AW performed literature search and drafted the manuscript. All authors were involved in treating the patients described and in the critical review of draft versions of the manuscript and approval of the final submission.

## Author Information

AW is a Specialty Registrar in Clinical Radiology, University Hospitals Bristol NHS Trust. MDK is a Consultant General Surgeon, North Bristol NHS Trust. LJ is a Consultant Vascular Interventional and General Radiologist, North Bristol NHS Trust.
